# *Sólo Se Vive Una Vez*: Evaluation of a Social Marketing Campaign Promoting HIV Screening and Prevention for Immigrant Latinxs

**DOI:** 10.1007/s10461-021-03165-4

**Published:** 2021-02-10

**Authors:** Harita S. Shah, Suzanne M. Dolwick Grieb, Alejandra Flores-Miller, Karine Yenokyan, Jimena Castellanos-Aguirre, Adena Greenbaum, Kathleen R. Page

**Affiliations:** 1 Department of Medicine, Johns Hopkins University School of Medicine, Baltimore, MD USA; 2 Department of Medicine, University of Chicago, Chicago, IL USA; 3 Department of Pediatrics, Johns Hopkins University School of Medicine, Baltimore, MD USA; 4 Baltimore City Health Department, Baltimore, MD USA; 5 Johns Hopkins University School of Public Health, Baltimore, MD USA

**Keywords:** Latinxs, Social marketing, HIV testing, HIV stigma, PrEP

## Abstract

Latinxs in the U.S. are disproportionately affected by HIV and more likely to have delayed diagnosis than their non-Latinx peers. We developed and implemented *Sólo Se Vive Una Vez* (You Only Live Once), the first Spanish-language campaign aimed at improving HIV testing and prevention among Latinx immigrants in Baltimore, Maryland. *Sólo Se Vive Una Vez* featured a website (www.solovive.org) and social marketing campaign promoting free HIV testing through the Baltimore City Health Department (BCHD) clinic and Latinx outreach team. The campaign was not associated with a change in the overall number of Latinxs obtaining HIV testing. However, Latinx HIV testers who reported being exposed to the campaign had significantly higher rates of high-risk sexual behaviors, mean number of sexual partners, and substance use. The campaign was also associated with increased PrEP referrals through the BCHD Latinx outreach team.

## Resumen

Los latinos en los Estados Unidos están desproporcionadamente afectados por el VIH y es más probable que sean diagnosticados más tarde que pacientes no latinos. Desarrollamos e implementamos Sólo Se Vive Una Vez, la primera campaña en español dirigida a mejorar la detección y prevención del VIH entre los inmigrantes latinos en Baltimore, Maryland. Sólo Se Vive Una Vez consiste en un sitio web (www.solovive.org) y una campaña en las redes sociales que promueve la prueba gratuita de VIH a través de la clínica del Departamento de Salud de la Ciudad de Baltimore así como la unidad móvil operada por nuestro equipo latino de promotores de salud. La campaña no se asoció con un cambio en el número absoluto de latinos que hicieron la prueba del VIH. Sin embargo, los latinos expuestos a la campaña que se hicieron la prueba del VIH tenían tasas más altas de conductas sexuales de alto riesgo, de parejas sexuales, y de uso de sustancias. La campaña también se asoció con un aumento de referidos a PrEP a través del equipo de promotores de salud latinos.

## Introduction

While considerable progress has been made to reduce the incidence of HIV in the United States (U.S.), Latinxs continue to be disproportionately affected by HIV [1]. Surveillance data from the Centers for Disease Control and Prevention (CDC) from 2012 to 2016 showed that the rates of HIV diagnoses decreased across all races except Latinxs, for whom the rates remained unchanged. Moreover, incidence increased for Latinx men who have sex with men (MSM) [1–3].

Of the estimated 263,000 Latinxs living with HIV in the U.S., approximately one fifth are unaware of their infection [2, 4]. Due to numerous barriers to HIV education and testing, Latinxs living with HIV present to healthcare with more advanced disease than their non-Hispanic peers [5–9]. According to CDC data, Latinxs born in Mexico and Central America have a higher incidence of HIV and approximately twice the risk for delayed diagnosis compared to U.S.-born Latinxs [7]. Additionally, foreign-born Latinxs have been shown to have disproportionately low awareness and uptake of pre-exposure prophylaxis (PrEP), an important means of HIV prevention, particularly for sexual and gender minorities [10–12]. Foreign-born Latinxs face specific structural and psychosocial barriers to HIV testing and care related to their immigration status. These include undocumented status, concern and possible fear of deportation, language discordance, lack of insurance, and difficulty navigating the U.S. healthcare system [13–16]. In particular, HIV stigma has been associated with reluctance to undergo testing among Latinxs and is a prominent barrier to HIV screening and linkage to care [13–16].

Stigma is a powerful discrediting force that perpetuates the cycles of structural and societal inequities. Parker and Aggelton argue that stigma inherently requires a differential of power (social, economic, or political) for a community to identify an undesirable attribute, construct stereotypes, and ultimately act on the stereotype by discriminating against the stigmatized [17]. Stigmatized groups including people living with HIV/AIDS are thus systematically disadvantaged in numerous structural ways including income, housing status, medical treatment and health [18]. Undocumented individuals are at the fringes of societal hierarchy, thereby compounding the propagation and perception of HIV stigma in a population who already faces structural barriers to care [13]. Research including our prior studies in Baltimore shows markedly high prevalence of stigmatizing beliefs, such as the association of HIV with “immoral” behaviors. High prevalence of community-level HIV stigma leads to a fear of social rejection in addition to fear of physical illness from HIV, both of which lead to a reluctance to undergo testing among Latinxs [13, 14, 19].

Consistent with nationwide trends, HIV-infected Latinxs in Baltimore, Maryland present with more advanced disease than their non-Hispanic white counterparts [20]. High levels of HIV stigma have been measured in Baltimore’s Latinx population and remain a key barrier to HIV testing [14, 19]. Early diagnosis and treatment are key to reducing HIV-associated morbidity and mortality and interrupting HIV transmission in the community and are the pillars of the HIV treatment as prevention strategy [21, 22]. To improve HIV testing services for Baltimore’s predominantly foreign-born Latinx population, the Baltimore City Health Department (BCHD) established a Latinx outreach team of bilingual community health workers or *promotoras* in 2008. The BCHD Latinx outreach team provided HIV/STI screening at venues with high concentrations of Latinx residents and businesses, including but not limited to: Latinx community-based organizations, clinics that predominantly serve Latinx patients, community fairs, bars, and parks. Testing was done via point of care HIV tests or in a mobile healthcare van in which phlebotomy services were available, based on patient preference and the logistics of the community-based venue. From its initiation in 2008 to July 2018, the BCHD Latinx outreach team provided HIV education and free testing to over 5000 Latinxs [23, 24]. However, few Latinxs were diagnosed with HIV through outreach services. In the year preceding this project, no Latinxs were diagnosed with HIV through outreach nor were Latinxs referred for PrEP, as those tested were not considered to be at substantial risk for acquiring HIV based on the CDC clinical practice guideline for PrEP [25]. Late diagnoses persisted among foreign-born Latinxs in Baltimore suggesting that outreach activities were not reaching those at highest risk of infection, including MSM [24].

Social marketing has been shown to be an effective strategy to promote HIV screening and target HIV stigma [26–28]. According to the 2019 Pew Hispanic Center survey, 70% of Latinxs use Facebook or other social networking sites [29]. Among Latinxs, marketing trends show that social media and internet use present effective means of expanding access to HIV testing and care [29, 30]. Social marketing campaigns have demonstrated promise in improving rates of HIV testing in Latinxs including among young Latinx immigrant MSM [31], heterosexually identifying Latinx men who have sex with men and women [32], and Latinxs living on the California-Mexico border [33]. Since HIV stigma is a known barrier to uptake of HIV testing, it is crucial that HIV testing campaigns are designed with stigma reduction in mind. Social marketing can be effective in reducing stigma by addressing misconceptions through education, breaking silence surrounding HIV by increasing visibility in the community and serving as a discreet venue for access to information and testing (as opposed to in person clinics) [26, 27].

To address the late diagnoses among Latinxs in Baltimore, we developed the *Sólo Se Vive Una Vez* campaign (hereafter referred to as the Vive campaign) in partnership with community partners, the Maryland Institute College of Art (MICA), Altavista Studios, and BCHD. The Vive campaign was based on the situated Information, Motivation, Behavioral Skills framework (sIMB) and the Transtheoretical Model (TTM) [34–36]. Based on research and iterative feedback from the community and stakeholders, the campaign initially targeted Latinx immigrant men and was then adapted to be more representative of Baltimore’s foreign-born Latinx community with particular attention to inclusion of sexual and gender minorities [36, 37]. The present analysis evaluates the effectiveness of *Sólo Se Vive Una Vez* with regard to the following outcomes: number of Latinxs obtaining HIV testing at BCHD, number of positive HIV tests, risk behaviors of Latinx HIV testers and number of Latinxs referred for PrEP.

## Methods

### The *Sólo Se Vive Una Vez* Campaign

The *Sólo Se Vive Una Vez* campaign ran June 22, 2018 to January 12, 2019 across Baltimore. It was developed with complementary individual and community level components, which have been shown to synergistically reduce HIV risk and improve testing and linkage to care [38–40]. The individual-level component of the campaign was a website (www.solovive.org) with culturally sensitive video modules individuals could select based on their perceived barrier(s) to HIV testing and/or HIV-related stigmatizing beliefs as identified in prior research [14, 19]. The website included an option for users to sign up for testing by entering their contact information, after which a BCHD Latinx outreach team member would contact them to arrange free HIV testing at a convenient outreach location or a BCHD STD Clinic. The website also included information on HIV prevention and PrEP, and it included a similar option for users to request PrEP. The website was designed to be accessible on mobile phones as Latinxs have the highest rates of accessing the internet and social media through mobile phones compared to other racial/ethnic groups, and cell phones have been demonstrated to be a highly acceptable method of receiving HIV-related health information among foreign-born Latinxs [41, 42].

The community-level components of the campaign included advertisements on social media (Facebook and Instagram), dating applications used by MSM (Grindr and Scruff), local Spanish-language radio stations, community outreach events, billboards, buses, newspapers, and posters. It is the combination of these components that was designed to reach high-risk individuals who previously were not being reached for testing, specifically: social media platforms to reach a wide audience and build recognizability, dating applications to reach MSM and individuals potentially engaging in high risk sexual activities, and in-person outreach and print materials at bars and street locations. The BCHD Latinx outreach team’s HIV testing events were coupled to the campaign and advertised on social media.

The advertisements featured messaging that addressed the most common stigmatizing beliefs and barriers to testing, as guided by prior focus groups and community surveys [14, 19]. An iterative process was used to identify then prioritize the highest performing platform and advertisements. The highest performing advertisements were Facebook advertisements that addressed the following reasons people defer HIV testing: “I am in a stable relationship,” “I am not at risk,” “I am scared of the result,” and “I am ashamed” (translated from Spanish) [43]. Given that an important driver of low uptake of PrEP among Latinxs is low awareness, advertisements also included education surrounding PrEP. In a prior study discussing the implementation and reach of the Vive campaign, we found that the Vive campaign reached over 84,592 people through social media advertisements and attracted 9,784 unique users to the website [43]. The majority of users were brought to the website via Facebook (6,881 users, 70.3%) and Grindr (2,097 users, 21.4%). In surveys completed by Latinx HIV testers at BCHD clinic and outreach, Facebook was identified as the most popular means of exposure to the campaign (by 43% of respondents) followed by the website (13%), community outreach events (12%), radio (9%), and other mediums (billboards, posters, buses, etc.).

### Measures

The present study measures the effectiveness of the Vive campaign via the primary outcomes of number of Latinxs obtaining HIV testing and positive HIV tests before and during the campaign. Secondary outcomes included risk behaviors of HIV testers and number of PrEP referrals.

### Primary Outcomes

The absolute number of Latinxs obtaining BCHD testing and number of positive HIV tests were chosen as the primary outcomes instead of testing rates because the denominator of the rate, i.e. the Latinx population in Baltimore, is thought to have changed significantly since the 2010 Census due to population migration. The numbers of Latinxs tested and the number of positive HIV tests in Baltimore City were obtained from BCHD surveillance data, which includes data from the Latinx outreach team and two sexual health clinics (East and West Baltimore) that provide free STD, HIV, HCV testing and treatment. Distributions of these variables were compared for the 7-month periods prior to the Vive campaign (November 6, 2017–June 21, 2018) and after initiation of the campaign (June 22, 2018–January 12, 2019), stratified by location of testing (clinic vs. outreach). Fisher’s exact test for proportions was used to evaluate the differences in these distributions using the Gmisc package in R.

### Secondary Outcomes

During the campaign (June 22, 2018-January 12, 2019), we recruited a convenience survey sample of patients receiving HIV testing services through the BCHD Latinx outreach team and the East Baltimore BCHD STD Clinic that is located close to high density Latinx neighborhoods. The brief Spanish-language survey included demographic information, if they had seen or heard of the Vive campaign and if the campaign influenced their decision to get tested for HIV. Eligible participants included Spanish-speaking adult (≥ 18 years old) patients who self-identified as Latinx and obtained an HIV test at the encounter. They were given self-administered paper surveys during registration at the clinic or outreach and were informed that the survey was voluntary and would not affect clinical care. The Institutional Review Board of Johns Hopkins Medicine approved this study.

Each survey was labeled with the respondent’s BCHD electronic medical record (EMR) number in order to link survey responses to risk assessments and HIV/STD testing history in the subject’s medical record. The BCHD standard risk assessment in the EMR includes: number of sexual partners in the past 3 months, gender of sexual partners, sexual behaviors in the past 12 months (vaginal sex, insertive sex, receptive sex, oral sex, sex without a condom, exchange of sex for drugs/money, sex with an anonymous person, sex with a person who is HIV + , and sex with a person who injects drugs), drug use in the past 12 months, and highest number of drinks on 1 occasion in the past month. High-risk sexual behavior was defined as reporting any of the following in the past 12 months: insertive anal sex, receptive anal sex, exchange of sex for drugs/money, sex with an anonymous person, sex with a person who is HIV + , or sex with a person who injects drugs in accordance with prior evidence [23, 24, 44]. Binge drinking in the past month was defined by the National Institute on Alcohol Abuse and Alcoholism (NIAAA) definition [45]. Given the high prevalence of history of sex without a condom in the past 12 months, we constructed a variable “condomless sex with risk” defined as history of condomless sex for people who were single with ≥ 1 sexual partner(s) in the past 3 months, or married or in a committed relationship with > 1 sexual partner in the past 3 months. PrEP awareness and patient referral for PrEP was recorded as part of the BCHD Latinx outreach form; it was not routinely documented at BCHD clinic visits, thereby limiting PrEP data to patients tested via outreach.

Trained research assistants entered survey responses and medical record data into a REDCap database [46]. Characteristics and risk behaviors of the population exposed to the campaign versus not exposed were compared using t-test and chi-squared analyses. All statistical analyses for survey data were conducted using Stata/SE Version 13.

## Results

### Primary Outcomes

#### Numbers of Latinxs Obtaining HIV Testing

During the pre-campaign and campaign periods, there were a total of 565 visits for HIV testing for Latinx adults ≥ 18 years old at the BCHD clinic and 1076 visits via BCHD Latinx outreach. There was no statistically significant difference in numbers of Latinxs tested before versus during the campaign at the BCHD clinic (278 vs. 287 testers) or outreach (548 vs. 528 testers) when comparing testers as a whole and when separated by gender.

#### HIV Outcomes

There was no statistically significant difference in the numbers of positive HIV tests before versus after campaign launch. In the BCHD clinic, there were 3 (1.1%) new HIV diagnoses before the campaign and 3 (1.0%) new HIV diagnoses during the campaign. In encounters from the BCHD Latinx outreach team, there were no new HIV diagnoses made before the campaign whereas there were 3 (0.6%) new HIV diagnoses made during the campaign (Fisher’s exact p = 0.12). An additional 3 patients had a prior diagnosis of HIV from a clinic outside BCHD but were out of care, and they were linked to care during the Vive campaign. For example, one patient was diagnosed at a private clinic and was not in care due to lack of insurance and high cost of the prescription; the patient saw the Vive campaign, entered his information on the website, and was linked to care obtaining antiretroviral therapy at BCHD.

### Secondary Outcomes

#### Risk Behaviors

Following campaign launch, from June 22, 2018 to January 12, 2019, we surveyed 427 Latinx adults obtaining HIV testing at the BCHD STD Clinic (n = 85, 19.9%) and through the BCHD Latinx outreach team (n = 342, 80.1%). The demographic characteristics and HIV testing history of participants are described in Table 1. Regarding campaign exposure, 31.6% (n = 135) of respondents reported having seen or heard of the Vive campaign, and 85.0% (n = 108) of respondents exposed to the campaign (25.3% of testers overall) reported that it influenced them to get tested. There were no statistically significant differences in demographics, self-identified sexual orientation, or HIV testing history of HIV testers exposed to the Vive campaign versus not exposed. Sexual orientation based on gender of patient and partners was also examined with minor differences: 1 self-identified heterosexual man had a male partner, 1 self-identified heterosexual man had male and female partners, and 7 self-identified heterosexual women had female partners. With sexual orientation defined in this manner, there remained no statistically significant differences between those exposed versus not exposed to the Vive campaign.

**Table 1:** Demographics and HIV testing history of respondents by exposure to *Sólo Se Vive Una Vez* campaign

	Overall		Not exposed to the vive campaign		Exposed to the vive campaign		
	N = 427	% or SD	N = 292	% or SD	N = 135	% or SD	
Age (mean, years)	35.6	10.2	35.6	10.3	35.6	9.9	t = 0.1, p = 0.928
Education							
Less than high school	309	72.4%	213	73.0%	96	71.1%	χ^2^ = 0.1, p = 0.743
High school graduate or higher	113	26.5%	76	26.0%	37	27.4%	
Did not respond	5	1.1%	3	1.0%	2	1.5%	
Country of origin							
Mexico	140	32.8%	97	33.2%	43	31.8%	χ^2^ = 9.2, p = 0.416
Honduras	93	21.8%	57	19.5%	36	26.7%	
El Salvador	89	20.8%	64	21.9%	25	18.5%	
United States	8	1.9%	5	1.7%	3	2.2%	
Other^a^	97	22.7%	69	23.6%	28	20.7%	
Gender							
Male	225	52.7%	157	53.8%	68	50.4%	χ^2^ = 2.1, p = 0.344
Female	197	46.1%	133	45.6%	64	47.4%	
Transgender Male	0	0.0%	0	0.0%	0	0.0%	
Transgender Female	5	1.2%	2	0.7%	3	2.2%	
Relationship status							
Single/Widowed	181	42.4%	117	40.1%	64	47.4%	χ^2^ = 2.2, p = 0.522
Married	128	30.0%	93	31.9%	35	25.9%	
Committed Relationship	109	25.5%	75	25.7%	34	25.2%	
Other	3	0.7%	2	0.7%	1	0.7%	
Did not respond	6	1.4%	5	1.7%	1	0.7%	
Prior HIV test							
No	114	26.7%	80	27.4%	34	25.2%	χ^2^ = 0.2, p = 0.653
> 1 year ago	191	44.7%	135	46.2%	56	41.5%	
< 1 year ago	95	22.3%	61	20.9%	34	25.2%	
Not sure	27	6.3%	16	5.5%	11	8.1%	

^a^Other countries of origin (reported by < 10% of participants): Guatemala, Ecuador, Dominican Republic, Peru, and Nicaragua

Latinx HIV testers with Vive campaign exposure had significantly more high-risk behaviors than those without campaign exposure as demonstrated in Table 2. 28.9% (n = 39) of HIV testers with Vive campaign exposure reported high-risk sexual behavior in the past 12 months compared to 18.5% (n = 54) of testers without campaign exposure (χ^2^ = 5.9, p = 0.016). HIV testers exposed to the Vive campaign had higher mean number of sexual partners in the past 3 months compared to those not exposed to the campaign (μ = 1.5 vs. 1.1 respectively, t = 2.2, p = 0.041). Condomless sex with risk was similarly high whether exposed or not exposed to the Vive campaign, reported by 64% and 68% of participants, respectively. There was a statistically significant difference in past 12-month substance use among HIV testers exposed to the campaign compared to testers without exposure (χ^2^ = 9.5, p = 0.025). Most notably, 5.9% (n = 8) of HIV testers with Vive campaign exposure reporting cocaine/crack use versus 1.4% (n = 4) of testers without exposure (χ^2^ = 7.0, p = 0.008).

**Table 2:** HIV risk factors and *Sólo Se Vive Una Vez* campaign exposure

	Overall		Not exposed to the vive campaign		Exposed to the vive campaign		
	N = 427	% or SD	N = 292	% or SD	N = 135	% or SD	
Sexual orientation							
Heterosexual	401	93.9%	274	93.8%	127	94.1%	χ^2^ = 2.8, p = 0.423
Bisexual	10	2.4%	7	2.4%	3	2.2%	
Homosexual	10	2.4%	8	2.7%	2	1.5%	
Did not respond	6	1.3%	3	1.0%	3	2.2%	
High-risk sexual behavior^a^ (Past 12 months)	93	21.8%	54	18.5%	39	28.9%	χ^2^ = 5.9, **p = 0.016**
Condomless sex with risk (Past 12 months)	137	32.1%	89	30.5%	48	35.5%	χ^2^ = 1.1, p = 0.296
Sexual partners (Past 3 months; mean (SD))	1.2	1.4	1.1	0.7	1.5	2.2	t = 2.2, **p = 0.027**
Substance use (Past 12 months)							
Marijuana	26	6.1%	17	5.8%	9	6.7%	χ^2^ = 9.5, **p = 0.025**
Cocaine/Crack	12	2.8%	4	1.4%	8	5.9%	
Crystal Meth	1	0.2%	0	0.0%	1	0.7%	
Other	1	0.2%	1	0.3%	0	0.0%	
Binge drinking (Past 1 month)	91	21.3%	63	21.6%	28	20.7%	χ^2^ = 0.1, p = 0.913

^a^In last 12 months, reports at least one of the following: insertive anal sex, receptive anal sex, exchange of sex for drugs/money, sex with an anonymous person, sex with a person who is HIV + , and sex with a person who injects drugs

#### PrEP

Of the 1076 patients tested by the BCHD Latinx outreach team, 772 (71.7%) had a PrEP assessment documented. Among these 772 patients, 95.2% (n = 735) had never heard of PrEP and only 4.8% (n = 37) reported knowledge of PrEP. After campaign initiation, the BCHD Latinx outreach team made 11 PrEP referrals based on patient’s risk profiles, in comparison to 0 referrals in the pre-campaign period (Fisher’s exact p < 0.001).

## Discussion

*Sólo Se Vive Una Vez* was the first social marketing campaign promoting HIV testing among foreign-born Latinxs in Baltimore, Maryland. The campaign was not associated with a change in overall number of Latinxs obtaining BCHD HIV testing, nor was it associated with a change in positive HIV tests among Latinxs. The campaign appeared to have reached Latinxs with high-risk sexual behaviors and more sexual partners. Bringing Latinxs with high-risk behaviors in contact with the BCHD Latinx outreach team presented an opportunity for HIV prevention, enabling PrEP referrals and education through outreach. Based on the results in Baltimore, the Maryland Department of Health decided to adopt the campaign to expand testing throughout the state of Maryland.

There was no statistically significant difference in the primary outcome of overall numbers of Latinxs obtaining testing at BCHD. It is important to learn from the null findings of our campaign and similar HIV social marketing campaigns to guide future research design and evaluation. Prior social marketing campaigns similarly demonstrated reach to high-risk populations without statistically significant change in overall HIV testing numbers. The *Hombre Sanos* campaign reduced rates of high-risk sexual behaviors among heterosexual Latinxs and Latinx men who have sex with men and women (MSMW), while HIV testing rates among heterosexually identified Latinx MSMW actually decreased following campaign implementation [32]. The *Tu Amigo Pepe* campaign led to an increase in testing rates in a cohort of 50 Latinx immigrant MSM, while HIV testing rates at the at the two sites promoted in the campaign did not change [31]. Systematic reviews of HIV prevention programs have shown that while social marketing campaigns have shown effectiveness in behavioral outcomes such as condom use, effects on HIV testing in Latinxs have been variable [47, 48]. This may reflect in part study design and in part the strengths and weaknesses of social marketing campaigns as a modality.

In terms of study design, the commonly used pretest–posttest design limits the strength of evaluation and there is a need for stronger quasi-experimental designs (acknowledging that randomized control trials are often not feasible) to better detect and characterize HIV testing outcomes [48, 49]. Though clinically important, HIV testing is a notably limited indicator of campaign effectiveness given that it is affected by numerous possible confounders (e.g., seasonal fluctuation, population migration) [33]. In terms of the potential of social marketing campaigns to impact HIV testing, prior campaigns have shown promise albeit the outcomes thus far have been mixed. Of note, efforts to improve HIV testing in Latinxs through home HIV self-testing and through targeting HIV stigma in church settings have been effective [50, 51]. It is possible that these venues are equally if not more efficacious in improving HIV testing for Latinxs than mass social marketing communications, which may be better suited for behavior change and risk reduction. Further research is required to guide what modality of intervention is best suited for specific groups within the diverse populations of Latinxs.

The Vive campaign appeared to have attracted Latinxs with high-risk behaviors to obtain HIV testing, particularly in the outreach setting. In the 10 years preceding the campaign, the BCHD Latinx outreach team had struggled to reach high-risk populations, and consequently had few positive HIV tests and PrEP referrals. In just 7 months following campaign implementation, the BCHD Latinx outreach team detected 3 positive HIV diagnoses and made 11 referrals for PrEP. The combination of messaging that included relatable individuals describing high-risk behaviors and getting tested, use of social media to expand reach, use of dating applications to reach individuals potentially engaging in high risk sexual activities, and in-person outreach at bars and street locations likely synergistically motivated individuals with high-risk behaviors to get tested. A noteworthy exception was the low numbers of Latinx MSM and gender minorities in our sample. Despite including input from LGBTQ Latinx community leaders in Vive development and distributing advertisements on dating apps and venues frequented by LGBTQ individuals, only 4.8% (n = 20) of HIV testers identified as homosexual or bisexual and 1.2% (n = 5) identified as transgender. Notably, the testing rate of high-risk populations like Latinx MSM is difficult to assess given that the stigma and homophobia faced by this population often leads to underreporting [52, 53]. Future interventions may better reach this population by including a youth focus or the use of home-based testing as these have increasing HIV testing among Latinx MSM in prior research [47].

In a prior study, we demonstrated that the Vive campaign achieved comparable rates of exposure (31.6%, n = 135) among HIV testers to other social marketing campaigns targeting HIV stigma and promoting HIV testing in Latinxs [27, 33, 43]. Moreover, the majority of testers exposed to the campaign (85.0%, n = 108) reported that it influenced them to get tested, acknowledging self-reported rates may be subject to social desirability bias. By coupling the reach of social marketing with the accessibility of community outreach, the Vive campaign reached HIV testers with high-risk behaviors that outreach events alone could not. Though our campaign did not change overall numbers of Latinxs obtaining HIV testing, our findings regarding risk profiles support prior research that social marketing may be an effective means of promoting HIV testing among Latinxs with high-risk behaviors [27, 31–33].

Furthermore, the Vive campaign promoted HIV prevention by facilitating PrEP education and referrals. Latinxs have been shown to have disproportionately low awareness and uptake of PrEP [10–12]. Only 4.8% (n = 37) of Latinx testers at BCHD outreach had heard of PrEP, a rate even lower than previously measured rates of awareness such as 8% among Puerto Rican MSM in a study by Dolezal et al. [10]. Through the combination of social marketing and community outreach services, the Vive campaign provided a means of reaching Latinx immigrants eligible for PrEP and connecting them with PrEP, while also providing education on PrEP to all testers.

Our study had noteworthy limitations. The majority of HIV testers captured by surveys were tested via the BCHD Latinx outreach team as BCHD clinic workflow limited the number of surveys completed, and this may have restricted our ability to measure and characterize Latinxs obtaining HIV testing at BCHD clinic (e.g., high-risk Latinxs who preferred the anonymity of presenting to clinic instead of outreach in their communities, or Latinxs with low literacy who were less able to complete the written survey or obtain outreach testing through the website or social media). Additionally, a small percentage of Latinxs exposed to the Vive campaign may have obtained testing from non-BCHD sources (less common given low insurance rates), and we did not measure changes in testers outside BCHD as we did not have access to the total number of tests in Baltimore. PrEP data was also only available for outreach encounters given differences in medical record documentation. While the procedures and resources for PrEP referrals through outreach were the same before and after the campaign, it is possible that the presence of the campaign affected the proactivity of the BCHD Latinx outreach team in discussing PrEP. Lastly, it is possible that the campaign had intangible effects, such as on perceptions of HIV in the Latinx community, that we were unable to measure.

## Conclusions

*Sólo Se Vive Una Vez* is Baltimore’s first Spanish-language campaign promoting HIV testing and addressing HIV stigma in the Latinx community. The overall numbers of Latinxs obtaining HIV testing were unchanged. However, the campaign appeared to attract Latinxs with high-risk behaviors to obtain HIV testing and receive access to PrEP. Based on the results of the campaign in Baltimore, the Maryland Department of Health chose to adopt the campaign to expand testing throughout the state. Social marketing campaigns may present an opportunity to promote HIV testing and PrEP uptake in specific underserved populations with high risk, but they must be paired with equitable and culturally competent access to HIV care and PrEP to lead to tangible improvements in population health.

## Data Availability

Data, materials, and code are available to qualified researchers upon request and with approval by the study team. Contact harita@uchicago.edu for any inquiries.
